# Development of a novel immunochromatographic lateral flow assay specific for *Mycobacterium bovis* cells and its application in combination with immunomagnetic separation to test badger faeces

**DOI:** 10.1186/s12917-017-1048-x

**Published:** 2017-05-12

**Authors:** Linda D. Stewart, Nuria Tort, Paul Meakin, Jose M. Argudo, Ruramayi Nzuma, Neil Reid, Richard J. Delahay, Roland Ashford, W. Ian Montgomery, Irene R. Grant

**Affiliations:** 1 Institute for Global Food Security, School of Biological Sciences, Queen’s University Belfast, Belfast, Northern Ireland UK; 2 Forsite Diagnostics Limited (now Abingdon Health), National Innovation Campus, Sand Hutton, York, UK; 3 Quercus, School of Biological Sciences, Belfast, Northern Ireland UK; 4 National Wildlife Management Centre, Animal and Plant Health Agency, Woodchester Park, Nympsfield, Gloucestershire, UK; 5 Animal and Plant Health Agency, Weybridge, New Haw, Addlestone, Surrey, UK

**Keywords:** *Mycobacterium bovis*, European badger (*Meles meles*), Faeces, immunomagnetic separation, Lateral flow device, Non-invasive test, Diagnostic specificity, Diagnostic sensitivity

## Abstract

**Background:**

The European badger is an important wildlife reservoir of *Mycobacterium bovis* implicated in the spread of bovine tuberculosis in the United Kingdom and Ireland. Infected badgers are known to shed *M. bovis* in their urine and faeces, which may contaminate the environment. To aid bovine tuberculosis control efforts novel diagnostic tests for detecting infected and shedding badgers are needed. We proposed development of a novel, rapid immunochromatographic lateral flow device (LFD) as a non-invasive test to detect *M. bovis* cells in badger faeces. Its application in combination with immunomagnetic separation (IMS) to detect *Mycobacterium bovis* cells in badger faeces is reported here.

**Results:**

A novel prototype LFD for *M. bovis* cells was successfully developed, with unique specificity for *M. bovis* and a limit of detection 50% (LOD_50%_) of 1.7 × 10^4^ *M. bovis* cells/ml. When IMS was employed to selectively capture and concentrate *M. bovis* cells from badger faeces prior to LFD testing, the LOD_50%_ of the IMS-LFD assay was 2.8 × 10^5^ *M. bovis* cells/ml faecal homogenate. Faeces samples collected from latrines at badger setts in a region of endemic bovine tuberculosis infection were tested; 78 (18%) of 441 samples tested IMS-LFD assay positive, whereas 140 (32%) tested IMS-qPCR positive (Kappa agreement −0.009 ± 0.044, *p* = 0.838). Subsequently, when 130 faeces samples from live captured, or captive, badgers of known infection status (on the basis of StatPak, interferon-γ and/or culture results) were tested, the IMS-LFD assay had higher relative diagnostic specificity (Sp 0.926), but poorer relative diagnostic sensitivity (Se 0.081), than IMS-qPCR (Sp 0.706, Se 0.581) and IMS-culture (Sp 0.794, Se 0.436).

**Conclusions:**

The novel IMS-LFD assay, although very specific for *M. bovis*, has low analytical sensitivity (indicated by the LOD_50%_) and would only detect badgers shedding high numbers of *M. bovis* (>10^4–5^ cells/g) in their faeces. The novel LFD would, therefore, have limited value as a non-invasive test for badger TB surveillance purposes but it may have value for alternative veterinary diagnostic applications.

## Background

*Mycobacterium bovis* is the causative agent of bovine tuberculosis (TB) in cattle, badgers and other wild and domestic mammals [1]. Bovine TB is the most serious endemic disease currently facing the livestock industry in the United Kingdom and Republic of Ireland [2]. Despite a systematic test and slaughter programme in cattle, which has been ongoing for many years within the United Kingdom, the incidence of bovine TB has continued to rise. This lack of success has been attributed in part to the presence of a reservoir of infection in the European badger (*Meles meles*) [3, 4]. Investigation of the dynamics of TB in wildlife and cattle herds using DNA fingerprinting of strains [5, 6] has revealed that badgers and cattle tend to have similar *M. bovis* spoligotypes, providing evidence of cross-species transmission [7]. In parts of the United Kingdom, herd incidence rates of TB in cattle more than doubled between 1998 and 2010, and although prevalence varies widely in badger populations, estimates of 6.3–33.9% infection (depending on county, overall 16.6%) were recorded in 2005 in endemic areas [3]. The ability to identify and monitor TB infection in the reservoir wildlife host may provide a valuable tool for targeting efforts to control risks of *M. bovis* transmission to cattle.

It is known that badgers can transmit *M. bovis* to cattle [8], but the precise route of transmission is unclear. A potential route if badgers and cattle come into close proximity is inhalation of aerosolised droplets; however, infected badgers are also known to shed *M. bovis* in their urine and faeces [9, 10], which may contaminate the environment. Badgers habitually defecate in clusters of shallow pits known as latrines which if accessible to cattle may act as a possible source of infection [11]. Infected badgers are known to intermittently shed *M. bovis* in their faeces in variable numbers. For example, King et al. [12] demonstrated heterogeneity in bacterial load for badgers in the Woodchester Park, Gloucestershire, study area, and reported shedding of 1 × 10^3^-4 × 10^5^ *M. bovis* cells per g of faeces. Faecal shedding is an indication of infectiousness, with shedding correlating with animals exhibiting more severe disease status [13]. *M. bovis* can persist in the environment for several months under certain conditions [14]. This environmental signal of the presence of *M. bovis* in a badger population was demonstrated by Courtenay et al. [15] who showed that the detectability of the bacterium at badger setts and latrines was strongly linked to the frequency of excretion detected in live-sampled badgers.

The *M. bovis* infection status of badgers in a given area may be assessed in various ways: by active surveillance involving the capture, anaesthesia and use of blood tests and microbiological culture of samples (invasive); by collecting and analysing badger faeces or other environmental samples (non-invasive); or, by the examination of badger carcasses after road traffic accidents (RTA) and tissue culture. The appropriate method to adopt depends on the question being asked and the geographical scale for which data are required. For example, RTA surveys have been used to provide coarse prevalence (or even presence/absence) data on a large geographical scale [16–19], whereas live capture and collection of clinical samples has been used to provide more detailed eco-epidemiological data over smaller areas [20]. The collection of faecal samples from latrines for TB surveillance in badgers has the potential to be used on a variety of geographical scales.

Since it is known that some infected badgers excrete *M. bovis* in their faeces [9, 10], we proposed the development of an immunochromatographic lateral flow device (LFD) and its use in combination with an existing immunomagnetic separation (IMS) technique [21, 22] as a non-invasive test for the presence of *M. bovis*. Immunochromatographic assays are easy to use, cheap to produce, and provide a rapid result (within 15 min), so are ideal for field use. The purpose of including IMS was to selectively capture and concentrate target mycobacterial cells from the faeces matrix before application to the LFD, to facilitate sample clean-up and improve detection sensitivity. Here, we report the successful development of a prototype *M. bovis*-specific LFD and its application in combination with IMS to test for evidence of *M. bovis* cells in badger faeces. We also assessed agreement between results of the field IMS-LFD assay and laboratory IMS-based methods (IMS-qPCR and IMS-culture) when faeces from badgers of unknown infection status were tested, and estimated relative diagnostic sensitivity (Se) and specificity (Sp) of the IMS-LFD assay by testing faeces from badgers for which we had independent information on their *M. bovis* infection status.

## Methods

### Bacterial cultures employed in the study

*Mycobacterium bovis* AF2122/97, *M. bovis* BCG NCTC 5692, *M. avium* subsp. *avium* NCTC 13034, *M. avium* subsp. *paratuberculosis* ATCC 19698, *M. fortuitum* NCTC 10394, *M. intracellulare* NCTC 10425, *M. kansasii* NCTC 10268, *M. xenopi* NCTC 10042, *M. terrae* NCTC 10856, *M. scrofulaceum* NCTC 10803, *M. marinum* NCTC 2275, *M. smegmatis* mc^2^ 155, *M. gordonae* NCTC 10267, *M. tuberculosis* H37Rv, and a field isolate of *M. hiberniae* were cultured in Middlebrook 7H9 broth containing 10% OADC supplement (both Difco) to stationary phase, harvested by centrifugation and washed in phosphate buffered saline, pH 7.2 (PBS, Sigma). Cell suspensions in PBS, containing 10^6^–10^7^ CFU/ml, were subjected to a 10 kGy dose of gamma radiation using a Gammabeam 650 cobalt irradiator, in order to kill the bacteria without damaging cell surface antigens and to render them safe to use in a Containment Level 2 laboratory. Irradiated PBS bacterial suspensions were stored at −80 °C until required. In addition, colonies of *M. tuberculosis* H37Rv grown on solid agar were emulsified in PBS and 80 μl tested directly on the LFD in a Containment Level 3 laboratory.

### Development of a prototype lateral flow device (LFD) specific for *M. bovis*

A selection of previously produced [21] or locally sourced *M. bovis*-specific monoclonal and polyclonal antibodies was tested for their suitability for incorporation into the proposed LFD. In order to select the most appropriate antibodies to serve as capture and detector reagents, a series of trials were carried out. All the antibodies were conjugated to gold nanoparticles and also all were immobilised at the Test (T) line of different nitrocellulose membranes at different concentrations. A cocktail of commercial anti-mouse-IgG IgG and anti-rabbit-IgG IgG was immobilized at the Control (C) Line position. A two-step wet assay was performed to evaluate recognition of *M. bovis* whole cells using the different combinations of membranes (with the immobilized antibodies in the T Line) and gold conjugates. The combination of antibodies and membrane which gave the strongest T-line was selected in order to produce a batch of working prototype devices to be evaluated.

### Assessment of detection specificity and cross-reactivity of the prototype LFD

PBS suspensions of the range of *Mycobacterium* spp. identified above and six different spoligotypes of *M. bovis* (SB140 (AF2122/97), SB129, SB273, SB142, SB263 and SB145) prepared as described above, were tested on the prototype LFD. Irradiated cultures were vortexed briefly before 80 μl were transferred to the sample well of the LFD. The presence or absence of a T-line on the LFD after 15 min at room temperature was assessed visually in each case.

### Determination of the limit of detection of the prototype LFD

The 50% limit of detection (LOD_50%_) of the LFD was determined by testing 10-fold serial dilutions of a stock of irradiated *M. bovis* AF2122/97 (containing 5.3 × 10^6^ CFU/ml) in PBS. Four replicate samples at each of four dilutions containing 10^5^, 10^4^, 10^3^ and 0 CFU/ml were tested. The presence or absence of a T-line was assessed both visually and using an LFD reader (Forsite Diagnostics Limited, York, UK) after the sample had been run on the LFD for 15 min at room temperature. The LOD_50%_ was determined using the generalized Spearman-Kärber LOD_50%_ calculation for 4-level spiking protocols [23].

### Coating of magnetic beads for immunomagnetic separation (IMS) and determination of *M. bovis* capture sensitivity and bead specificity

The MyOne tosylactivated Dynabeads (Life Technologies, Paisley, UK) previously used by Stewart et al. [22] for immunocapture of *M. bovis* from bovine lymph node tissue are 1 μm in diameter. These proved to be too large to run along the LFD, so smaller carboxylated magnetic beads, available in three sizes (200, 300 and 500 nm) were sourced (Ademtech, France). All three sizes of Ademtech bead were coated with the *M. bovis*-specific 11G3 monoclonal antibody and EEA302 biotinylated peptide [21] separately, and also with a mixture of the peptide and the antibody (dually coated), using the Carboxyl-Adembeads Coupling Kit (Ademtech 02820) according to the manufacturer’s instructions. Tenfold serial dilutions (10^−1^ to 10^−5^) of irradiated *M. bovis* AF2122/97 (10^6^–10^7^ CFU/ml) were prepared and 1 ml of each dilution was subjected to automated IMS using the Dynal BeadRetriever (Life Technologies) (21) using the different sized/coated Adembeads, with resuspension of the beads after IMS in 100 μl Tris-EDTA (TE) buffer. After extraction of DNA by heating at 100 °C for 25 min, the samples were analysed using *M. bovis* touchdown PCR [24] and visualised using agarose gel electrophoresis PCR, as described in Stewart et al. [21]. As controls, and to enable comparison of capture sensitivities achieved, dually coated MyOne Tosylactivated Dynabeads (Life Technologies) and a dilution series of irradiated *M. bovis* AF2122/97 in TE buffer not subjected to IMS were included.

Specificity of the 200 and 300 nm coated beads was assessed by IMS experiments involving the range of *Mycobacterium* spp. described above. Each species was tested on two separate occasions. Stationary phase broth cultures of each species were diluted to 10^3^–10^4^ CFU/ml in Middlebrook 7H9/OADC broth. A 100 μl sample of the dilutions was spread onto Middlebrook 7H10/OADC agar plates to determine CFU/ml before IMS and 1 ml of each dilution was then subjected to automated IMS using each of the coated beads. Following IMS, beads were resuspended in 1 ml Middlebrook 7H9/OADC broth (maintaining original sample volume in order to permit direct comparison of colony counts before and after IMS), 100 μl of which was spread onto Middlebrook 7H10/OADC agar plates. Agar plates were incubated at 30, 37 or 42 °C (as appropriate for the *Mycobacterium* sp. concerned) until colonies were evident. Mean colony count following IMS was expressed as a percentage of the number of CFU present in the original suspension before IMS to calculate the degree of non-specific binding by *Mycobacterium spp*. other than *M. bovis.*

### Optimisation of faeces sample preparation protocol

Optimisation of the faeces sample preparation protocol is described in detail elsewhere [25]. Briefly, a number of factors in relation to application of IMS in the field needed to be considered before arriving at a finalised field IMS protocol. These included the volume of diluent required to homogenise the faecal samples, the effect of the faecal matrix on bead retrieval, the time needed to retrieve the beads, the dilution factor required, the quantity of beads required, the assessment of matrix effect on capture of *M. bovis* by coated beads, and the effect of nylon strainers on the recovery of *M. bovis*.

### Limit of detection of IMS-LFD assay applied to faeces

The LOD_50%_ of the IMS-LFD assay was determined twice using two different *M. bovis* negative badger faeces samples as the spiking matrix. Four replicate samples were spiked at four cell concentrations (10^5^, 10^4^, 10^3^ and 0 CFU/ml) before the field IMS-LFD assay was applied. The presence or absence of T-lines was assessed visually. The LOD_50%_ was, once again, estimated using the generalized Spearman-Kärber LOD_50%_ calculation for 4-level spiking protocols [23].

### Ability of the novel IMS-LFD assay to detect *M. bovis* in naturally infected badger faeces

Badger faeces samples were collected from latrines at 110 setts throughout Northern Ireland. A random sample of 1 km squares containing main setts, located during the Northern Ireland Badger Survey 2007/08 [26], were selected to be visited for sampling purposes. These setts were located in geographic areas with high, moderate and low reported incidence of tuberculosis breakdowns in cattle, based on information supplied by the Department of Agriculture and Rural Development for Northern Ireland. On arrival at each main sett, a single latrine was located from which up to a maximum of five of the freshest appearing faecal samples were collected; each sample was taken from a different dropping.

**Fig. 1:**
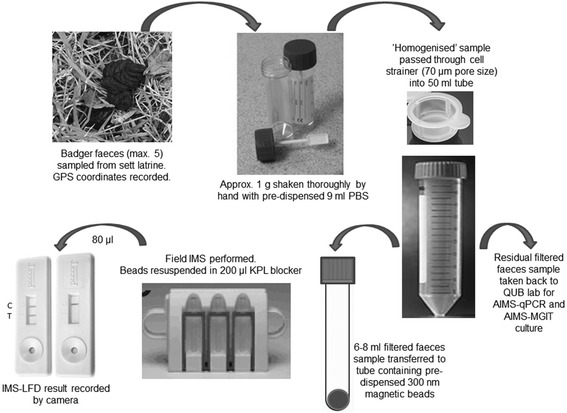
Schematic outlining the field IMS-LFD testing procedure for testing badger faeces

The IMS-LFD procedure was performed in the field as summarised schematically in Fig. 1. Approximately 1 g of badger faeces was transferred to a tube containing 9 ml PBS pH 7.2 and the sample was shaken vigorously by hand. When a homogeneous suspension was obtained, the sample was filtered through a 70 μm cell strainer (Falcon) into a 50 ml centrifuge tube. A sub-sample of 6–8 ml of this homogenised, filtered faeces sample was poured into a tube containing 20 μl of antibody- and peptide- dually coated 300 nm carboxylated magnetic beads. The sample was incubated at ambient temperature for 30 min and shaken every 5 min. The tube was placed in a DynaMag™-15 magnetic rack (Life Technologies) for 10 min before the supernatant was carefully poured off, leaving the beads behind. Beads were washed three times by shaking in approx. 5 ml PBS-0.05% Tween 20 (PBS-T, Sigma) per wash with separation for 2 min on the magnetic rack between washes. After the third wash the beads were resuspended in 200 μl Detector™ block solution (KPL Inc., Gaithersburg, USA) and 80 μl was transferred to the test well of the LFD. After 15 min the IMS-LFD result was recorded as ‘positive’ if both Control (C) and Test (T) lines were visible or ‘negative’ if only the C line was observed. Digital photographs were taken to record test outcomes. The LFDs were retained and returned to the laboratory where the presence of a T line was subsequently verified using a Forsite LFD reader. Residual bead suspensions were also returned to the laboratory for Ziehl-Neelsen staining and examination by light microscopy, in order to verify the presence of large numbers of acid-fast cells in samples which had tested IMS-LFD positive in the field.

### Laboratory IMS-based tests on residual faecal homogenates

The residual 1–2 ml portion of each homogenised, filtered badger faecal sample was returned to the laboratory where automated IMS followed by real-time qPCR (IMS-qPCR) and MGIT™ culture (IMS-culture) were carried out. One ml of each homogenised, filtered badger faecal sample was subjected to automated IMS with dually antibody- and peptide-coated MyOne tosylactivated Dynabeads [21] using a Dynal BeadRetriever (both Life Technologies). Following automated IMS, the beads plus any captured *M. bovis* cells were resuspended in 500 μl Middlebrook 7H9 broth which was then split between a 100 μl sample for qPCR (method of Sweeney et al. [27]) and 400 μl for BACTEC™ MGIT™ culture (Becton Dickinson). DNA was released from bead-bound *M. bovis* cells by boiling for 25 min and then purified by Zymoclean columns (ZymoResearch) before qPCR was performed using an Eco-PCR instrument (Illumina, Inc.). The reaction volume was 25 μl comprising 12.5 μl of TaqMan Gene expression ×2 master mix (Life Technologies), 1 μl (20 pmol/μl) forward RD4 flanking primer (5′ TGTGAATTCATACAAGCCGTAGTCG 3′), 1 μl (20 pmol/μl) reverse RD4 flanking primer (5′ CCCGTAGCGTTACTGAGAAATTGC 3′), 1 μl (10 pmol/μl) RD4 hydrolysis probe (5′ 6-FAM–AGCGCAACACTCTTGGAGTGGCCTAC–MGB 3′), 7 μl of nuclease-free sterile water and 2.5 μl of template DNA. The RD4 hydrolysis probe and qPCR primers were purchased from Life Technologies. Reaction conditions were: 50 °C for 2 min, 95 °C for 10 min, and 40 cycles of 95 °C for 15 s and 60 °C for 1 min. Duplicate 2.5 μl aliquots of DNA were tested for each faecal sample. Both aliquots had to report positive or negative for a definitive IMS-qPCR result to be recorded. In instances where there was disagreement between the duplicate results for any sample, the qPCR was repeated. If disagreement between duplicate results still existed after the second PCR then the sample was recorded as IMS-qPCR negative (interpretation adopted by Travis et al. [28]). A six point standard curve, generated using DNA from a dilution series (10^6^–10 CFU/ml) of irradiated *M. bovis* AF2122/97, and a no template control (water only), were run in duplicate in each qPCR run.

For IMS-culture, after IMS the beads (400 μl) were inoculated directly into BACTEC™ MGIT™ culture tubes supplemented with 10% OADC supplement and PANTA antibiotic supplement (all Becton Dickinson). The MGIT cultures were observed visually (because no MGIT 960 instrument was available in the laboratory) at intervals over an incubation period of up to 12 weeks at 37 °C for signs of growth (turbidity change). After 4, 8 and 12 weeks of incubation 100 μl of each MGIT culture showing evidence of growth was removed, boiled at 100 °C for 25 min and checked for presence of *M. bovis* DNA by Touchdown PCR targeting the IS6110 element and employing INS1 and INS2 primers [24]. The Touchdown PCR was able to detect down to 50 *M. bovis* CFU/ml (Stewart and Grant, unpublished data). An IMS-culture positive result was declared if MGIT cultures showing evidence of growth (turbidity) tested Touchdown PCR positive. If no growth was observed after 12 weeks or if the final Touchdown PCR results for the MGIT positive cultures at 12 weeks were all negative, then a negative IMS-culture result was declared.

### Assessment of the diagnostic specificity and sensitivity of the IMS-LFD assay by testing faeces from badgers of putative known infection status

In total, 130 faecal samples were collected from badgers for which independent live animal TB diagnostic test results were also available. The samples were tested by the IMS-LFD assay, IMS-qPCR and IMS-culture, as described above for the badger faecal samples collected from latrines, except that sample preparation and the IMS-LFD test were performed in the laboratory. Of the 130 faecal samples, 100 had been obtained, following administration of an enema, from badgers trapped as part of the long-term capture-mark-release project at Woodchester Park, Gloucestershire, England. In order to maximise the likelihood that a proportion of these samples came from *M. bovis-*infected animals, we targeted collection at badger social groups with evidence of current or recent live animal test positive results. Following collection all faeces samples were stored at -70 °C for up to 7 months prior to testing. BrockTB Stat-Pak® (Chembio Diagnostic Systems, Inc., Medford, NY), culture (of sputum, faeces, urine, wound/abscess swabs), and interferon-gamma enzyme immunoassay (IFN-γ EIA [29]) test results, obtained over the previous 1–2 years, were available for these animals. The remaining 30 faecal samples were obtained from captive badgers that were originally trapped in a part of the United Kingdom with very low incidence of TB in cattle. These 30 badgers had tested negative for *M. bovis* infection by the IFN-γ EIA test and bacteriological culture of clinical samples (sputum, urine and rectal swab) on three sequential occasions over a three month period. They were, therefore, considered putative ‘non-infected’. All 130 faeces samples were blind tested by staff at Queen’s University Belfast.

### Statistical analysis

Descriptive statistics captured the prevalence of *M. bovis* positive results using the three IMS-based tests (IMS-LFD, IMS-qPCR and IMS-culture) on 441 badger faeces of unknown disease infection status collected in Northern Ireland, and 130 badger faecal samples collected from Woodchester Park (*n* = 100) and captive (*n* = 30) animals for which contemporaneous or previous live animal diagnostic test results were available. Venn diagrams were prepared to illustrate inter-relationships between the results obtained with each test. Cross-tabulation of results permitted determination of Kappa statistics, as a measure of the agreement between test results, which were interpreted according to Landis and Koch [30]. McNemar’s test was performed on 2 × 2 contingency tables of results for the three IMS-based tests versus the badgers’ putative TB infection status derived on the basis of the live animal diagnostic test results, to permit estimation of diagnostic sensitivity (Se) and specificity (Sp) of the field IMS-LFD, IMS-qPCR and IMS-culture tests applied to faeces. Since no gold standard diagnostic test for *M. bovis* in badgers exists, estimates of diagnostic specificity and sensitivity are relative to the live animal tests applied. All statistical analyses were performed using SPSS 21 (IBM), unless otherwise stated.

## Results

### Development of a prototype *M. bovis*-specific LFD

A specific monoclonal mouse anti-*M. bovis* antibody (Mab) and a rabbit anti-*M. bovis* polyclonal antibody (Pab) were successfully conjugated to the gold nanoparticles, and the resulting conjugates were found to be stable. Potential capture reagents were immobilised at the T-line position on the membranes and a cocktail of commercial anti-mouse-IgG and anti-rabbit-IgG IgG was immobilized at the C-line position. Following trials with different combinations and concentrations of capture and detector reagents, it was concluded that the working prototype LFD for the detection of *M. bovis* whole cells was comprised of ‘membrane 1’ with polyclonal antibody immobilised at the T-Line to act as the capture reagent and Mab conjugated to gold nanoparticles to act as the detector reagent. A batch of 1000 prototype LFDs was produced by Forsite Diagnostics Ltd., York, for evaluation at Queen’s University Belfast. For commercial reasons, no additional detail about the membrane or antibodies used for the prototype LFD device can be provided here.

**Fig. 2:**
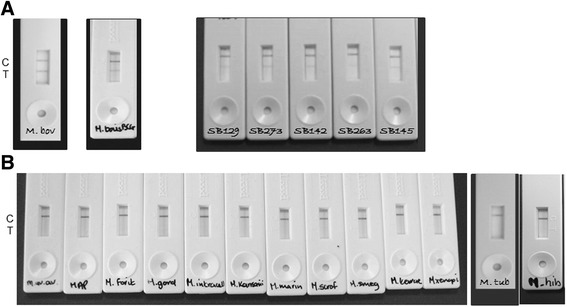
Outcomes of specificity testing of the prototype lateral flow immunochromatographic device (LFD) demonstrating (**a**) the presence of a positive T-line with *Mycobacterium bovis* AF2122/97, *M. bovis* BCG NCTC 5692 and five other *M. bovis* spoligotypes and (**b**) only the presence of a C-line for all other *Mycobacterium* spp. tested

When the detection specificity of the prototype LFD was evaluated by testing a range of different *Mycobacterium* spp. diluted in PBS, the prototype LFD yielded a positive T-line for *M. bovis* (all six spoligotypes tested) and *M. bovis* BCG only (Fig. 2a), and gave a negative T-line with all other *Mycobacterium* spp. tested (Fig. 2b). In terms of detection sensitivity, the LOD_50%_ for *M. bovis* suspended in PBS was determined to be 1.7 × 10^4^ *M. bovis* cells/ml.

### IMS modification

Evaluation of the smaller Ademtech carboxylated magnetic beads indicated that, like the previously used MyOne Tosylactivated beads, dually coated beads (i.e. coated with both peptide and IgM antibody) produced the best *M. bovis* capture results. The dually coated 300 nm and 200 nm beads showed similar capture capability from both buffer and spiked faeces, with <3% non-specific binding with all the other non-target mycobacteria tested. It was, therefore, concluded that either sized bead could be used with the LFD, and we decided to use the 300 nm beads.

### Combining field IMS with the LFD

After field IMS the beads were resuspended in 200 μl PBS for analysis on the LFD. However, it was found that uncoated beads, coated beads and the storage buffer recommended by Ademtech all produced a false positive T-line on the LFD after IMS. To resolve this issue an extra step of quenching the activated unused carboxyl groups present on the bead surface using ethanolamine was introduced during coating of the beads, and various blocking solutions were investigated as alternatives to the Ademtech storage buffer for resuspension of the coated beads. The false positive issue was resolved by resuspension of beads after IMS in KPL Detector™ block, a commercially available blocking solution (KPL Inc., Gaithersburg, Maryland). The finalised field IMS-LFD protocol was as illustrated in Fig. 1.

The LOD_50%_ of the field IMS-LFD was estimated to be 2.8 × 10^5^ *M. bovis* cells/ml faeces homogenate (1:10 dilution), so the detection sensitivity of the LFD was reduced when IMS preceded LFD detection, and faeces homogenates rather than PBS suspensions were being tested.

### Capability of the IMS-LFD test to detect *M. bovis* in badger faeces relative to the laboratory-based IMS-qPCR and IMS-culture tests

**Fig. 3:**
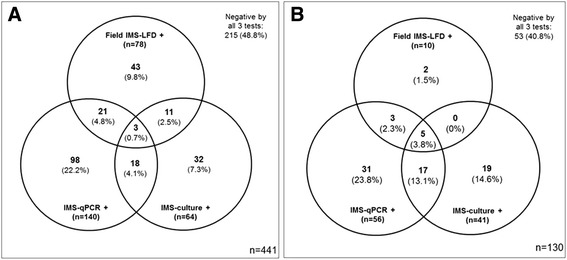
Venn diagrams showing the numbers (percentage) of faeces samples that tested positive by field IMS-LFD, IMS-qPCR and IMS-culture from 441 badgers of unknown infection status collected from latrines at main setts in Northern Ireland (**a**) and from 100 live captured badgers and 30 captive putative TB negative badgers for which independent live animal diagnostic test data were available (**b**). Areas of overlap indicate positive test results in common

A total of 441 badger faecal samples, collected from 110 main setts throughout Northern Ireland were tested. A mean of four faeces samples were collected from a single latrine at each main sett. Using the individual faecal sample as the unit of analysis, 78 faecal samples (18%) tested positive with the field IMS-LFD test, 140 (32%) tested positive by IMS-qPCR, and 64 (15%) tested positive by IMS-culture, with only three faecal samples (0.7%) testing positive by all three tests (Fig. 3a). Given the LOD_50%_ of the IMS-LFD assay applied to badger faeces indicated above, a positive result with this assay should indicate the presence of ~10^5^ *M. bovis* cells/ml, which should be visible microscopically. Acid-fast bacteria attached to the magnetic beads were observed for several of the IMS-LFD positive faecal samples, providing confirmation that high numbers of acid-fast bacteria (assumed to be *M. bovis*) were indeed present when an IMS-LFD positive result was obtained.

Field IMS-LFD results were compared with results of the two laboratory IMS-based methods; neither of which was being considered as the gold standard method for detecting *M. bovis* in faeces. There was no significant association between the results of field IMS-LFD, IMS-qPCR or IMS-culture (*P* > 0.05). Kappa statistics indicated ‘poor’ agreement between IMS-LFD and IMS-qPCR results (Kappa = −0.009, 95% CI: -0.095 to 0.077, *p* = 0.838) and ‘slight’ agreement (Kappa = 0.045, 95% CI: -0.054 to 0.144, *p* = 0.342) between IMS-LFD and IMS-culture results (Table 1A).

**Table 1:** Cross-tabulation of field IMS-LFD, IMS-qPCR and IMS-culture results for 441 badger faecal samples freshly collected from latrines at main setts in Northern Ireland (A), and 130 faecal samples obtained from live captured or captive badgers of putative known TB infection status (B)

(A)						
Field test result	Laboratory-based test results:					
	IMS-qPCR +	IMS-qPCR -	Kappa ± SE (significance)	IMS- culture +	IMS- culture -	Kappa ± SE (significance)
IMS-LFD +	24	54	−0.009 ± 0.044	14	64	0.045 ± 0.051
IMS-LFD -	116	247	(*p* = 0.838 ^NS^)	50	313	(*p* = 0.342^NS^)
Total	140	301		64	377	
(B)						
Field test result	Laboratory-based test results:					
	IMS-qPCR +	IMS-qPCR -	Kappa ± SE (significance)	IMS- culture +	IMS- culture -	Kappa ± SE (significance)
IMS-LFD +	8	2	0.129 ± 0.056	5	5	0.083 ± 0.063
IMS-LFD -	48	72	(*p* = 0.014*)	36	84	(*p* = 0.083^NS^)
Total	56	74		41	89	

^NS^ no significant association,﻿ * *P*<0.05, significant association at the 95% level

### Estimation of the relative diagnostic specificity and sensitivity of the IMS-LFD assay by testing faeces from badgers of known infection status

The TB infection status of the 100 badgers from Woodchester Park that had contributed the faecal samples tested was only revealed to laboratory staff once results became available for the IMS-based tests. The badgers were categorised as putative ‘TB infected’ if a positive result had been obtained for Stat-Pak®, culture or IFN-γ EIA tests on any test occasion previously, and as putative ‘non-infected’ if a negative result had been obtained for all three live animal tests on each previous testing occasion. Overall, there were 62 faecal samples from putative ‘TB infected’ and 38 from putative ‘non-infected’ badgers in this cohort. The field IMS-LFD tested positive for 10% of faecal samples obtained by enema from these 100 live captured badgers, whilst IMS-qPCR and IMS-culture tested positive for 56% and 41% of samples, respectively. Five (3.8%) samples tested positive by all three IMS based tests (Fig. 3b). Two samples tested positive by the IMS-LFD test and not by either of the other two IMS-based tests. All of the 30 faeces from putative ‘non-infected’ captive badgers tested negative by field IMS-LFD, IMS-qPCR and IMS-culture, so no false positive IMS-LFD results were obtained. Overall, Kappa statistics indicated ‘poor’ agreement between IMS-LFD and IMS-qPCR results (Kappa = 0.129, 95% CI: 0.019 to 0.238, *p* = 0.014) and also between IMS-LFD and IMS-culture results (Kappa = 0.083, 95% CI: -0.056 to 0.221, *p* = 0.083) for the 130 faeces samples tested (Table 1B).

Cross-tabulation of badger infection status (on the basis of prior live animal diagnostic test results) with IMS-LFD, IMS-qPCR and IMS-culture results (Table 2) enabled estimates of relative diagnostic specificity (Sp) and sensitivity (Se) to be obtained using McNemar’s test. IMS-qPCR and IMS-culture had much higher diagnostic Se (0.581 and 0.436, respectively) than the IMS-LFD test (0.081) (Table 2). However, the IMS-LFD test had greater diagnostic Sp (0.926) than either IMS-qPCR (0.706) or IMS-culture (0.794) (Table 2).

**Table 2:** Estimates of the diagnostic specificity (Sp) and sensitivity (Se) of the field IMS-LFD and laboratory-based IMS-qPCR and IMS-culture tests for *M. bovis* in faeces from 100 live captured badgers and 30 live captive badgers relative to live diagnostic test results, obtained using McNemar’s test (95% confidence intervals are indicated in parentheses)

Test result	Putative TB infected	Putative Non-infected	Total	Diagnostic sensitivity, Se	Diagnostic specificity, Sp
IMS-LFD +	5	5	10	0.081	0.926
IMS-LFD -	57	63	120	(0.027–0.178)	(0.837–0.976)
	62	68	130		
IMS-qPCR +	36	20	56	0.581	0.706
IMS-qPCR -	26	48	74	(0.449–0.705)	(0.583–0.811)
	62	68	130		
IMS-culture +	27	14	41	0.436	0.794
IMS-culture -	35	54	89	(0.310–0.567)	(0.678–0.882)
	62	68	130		

## Discussion

The IMS-LFD test described here was developed with a view to it being a rapid and non-invasive test to detect the presence of whole *M. bovis* cells in badger faeces. To our knowledge, the prototype LFD developed is the first of its kind. Other *M. bovis* LFDs are available commercially, but these detect either serum antibodies to *M. bovis* (BrockTB Stat-Pak® assay or DPP® CervidTB assay, both Chembio Diagnostic Systems, Inc., Medford, NY), or the MPT64 antigen secreted by members of the *Mycobacterium tuberculosis* complex, which includes *M. bovis*, in liquid culture (BD MGIT™ TBc Identification Test, Becton, Dickinson and Company, NJ; SD Bioline TB Ag MPT 64, Standard Diagnostics, Inc., Gyeonggi-do, Republic of Korea; Capilia TB-Neo kit, TAUNS Laboratories, Inc., Shizuoka, Japan). The unique specificity of the LFD developed during this study is due to the particular monoclonal antibody conjugated to gold nanoparticles and used as the detector reagent in the novel LFD; for commercial reasons, no further details of this antibody can be provided. The novel *M. bovis*-specific LFD was used in combination with a modified IMS technique (involving smaller magnetic beads) in order to capture and concentrate *M. bovis* cells from the faeces matrix and remove gross faecal components that could potentially block movement of the beads along the LFD.

When 441 badger faeces samples collected from latrines at main setts throughout Northern Ireland were tested by the IMS-LFD assay, IMS-qPCR and IMS-culture, *M. bovis* was detected in 18%, 32% and 15% of faecal samples respectively (Fig. 3a). There was little agreement between the results of the three IMS-based tests. Whilst the same faecal homogenate was tested by all three methods, different volumes of material were tested in the field (by IMS-LFD) and in the laboratory (by IMS-qPCR and IMS-culture). Automated IMS in the laboratory was restricted to analysing 1 ml of sample whereas the field IMS was carried out on a 6–8 ml volume of filtered faecal homogenate. We estimate that there was the equivalent of ~250 mg faeces per 80 μl bead sample applied to the LFD after IMS in the field, ~5 mg faeces per IMS-qPCR reaction, and ~80 mg faeces per IMS-culture (assuming no losses during sample processing), so the higher volume of sample used for the IMS-LFD assay may explain the few extra samples which tested positive by the field IMS-LFD test (Fig. 3a). That said, we estimated in preliminary studies [25] the LOD_50%_ of IMS-qPCR to be 1.7 × 10^4^ *M. bovis* cells/ml of faeces homogenate (or 1.7 × 10^5^ *M. bovis* cells/g faeces), indicating that the IMS-qPCR method should theoretically have greater detection sensitivity than the field IMS-LFD assay (which was found to have an LOD_50%_ of 2.8 × 10^5^ *M. bovis* cells/ml of faecal homogenate or 2.8 × 10^6^ *M. bovis* cells/g faeces). This being the case, more *M. bovis* positive faecal samples should have been detected by IMS-qPCR than by IMS-LFD, which was the situation during this study. Furthermore, differently sized magnetic beads were used in the field and laboratory tests, because only the smaller (300 nm) coated beads would pass along the LFD. Despite being coated with the same *M. bovis*-specific binders, we know that the 300 nm Ademtech beads have slightly less capture capability than the 1 μm MyOne Tosylactivated Dynabeads; this may also have influenced results obtained for the field IMS-LFD test compared to the laboratory IMS-based tests. We also suspect, but cannot demonstrate, that various environmental, logistical and practical considerations may have influenced the faecal sample preparation and IMS capture steps in the field, potentially leading to non-optimal performance of the IMS-LFD test. Some or all of these factors may have contributed to, and hence explain, the lack of agreement between field IMS-LFD and laboratory IMS-based test results. Test results for badger faeces collected from latrines at setts have demonstrated for the first time that the novel LFD is capable of detecting *M. bovis* cells in naturally contaminated badger faeces. However, due to the lack of agreement between results of the different IMS-based tests (Table 1A), with the lowest number of *M. bovis* positive samples being detected by IMS-LFD, results suggest that the novel IMS-LFD assay would be of limited use for badger TB surveillance purposes. Although the LFD was shown to have unique specificity for *M. bovis* (Fig. 2), the analytical sensitivity indicated by the LOD_50%_ of the combined IMS-LFD assay (2.8 × 10^5^ *M. bovis* cells/ml faecal homogenate) means that only badgers shedding high numbers of *M. bovis* in their faeces would be detected, and infected badgers shedding lower numbers of *M. bovis* would test negative by the IMS-LFD assay. Given its reasonably high LOD_50%_, the novel IMS-LFD test may be more suited for applications where high numbers of *M. bovis* would be encountered, such as confirming the isolation of *M. bovis* from animal tissues in liquid cultures in the veterinary diagnostic laboratory context. This possibility is currently being investigated.

The ability of a novel diagnostic test to correctly identify infected and non-infected animals is an important consideration, so in order to determine the relative diagnostic sensitivity and specificity of the novel IMS-LFD assay, we tested 130 faeces samples from badgers for which independent diagnostic test results were available. Of these, the IMS-LFD tested positive in 10 (7.7%) cases, whilst 56 (43.0%) and 41 (31.5%) samples tested positive by IMS-qPCR and IMS-culture, respectively (Fig. 3a). Test results for the subset of 30 faeces samples from putative TB negative captive badgers indicated that all samples were IMS-LFD, IMS-qPCR and IMS-culture negative; thus providing evidence of the high relative specificity of the novel IMS-LFD test, and indeed the other two IMS-based tests. The IMS-LFD test was found to have high diagnostic Sp (0.926) but low diagnostic Se (0.018), relative to the comparator live animal diagnostic tests, indicating an inability to detect low numbers of *M. bovis* cells in faeces, whereas IMS-qPCR and IMS-culture had lower diagnostic Sp (0.706 and 0.794, respectively) but higher diagnostic Se (0.581 and 0.436, respectively). It is acknowledged that potential mis-classification of the TB infection status of the badgers in our study due to false negative live animal test results may have impacted our estimates of the sensitivity and specificity of IMS-based tests (Table 2), so the latter should be treated with caution and only as relative measures of test performance.

**Fig. 4:**
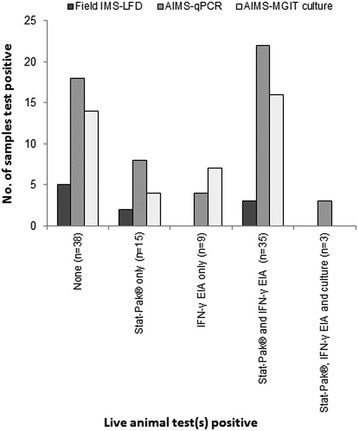
Relationships between the IMS-based test results for 100 faeces samples from badgers in the Woodchester Park study area and the *M. bovis* infection status of these badgers as indicated by results of contemporaneous or previous Stat-Pak®, culture and Interferon-gamma tests

The percentages of badger faeces samples from Woodchester Park testing *M. bovis* positive by IMS-qPCR and IMS-culture reported here appear relatively high. However 62 of the 100 faeces samples we tested were from putative ‘TB infected’ badgers with at least one previous positive live animal test result. Hence the 8.1%, 58% and 43.5% which tested IMS-LFD, IMS-qPCR and IMS-culture positive, respectively, could be considered as the percentage of *M. bovis* faecal positives amongst putative ‘TB infected’ badgers only. However, it is notable that the presence of *M. bovis* was also indicated by one or more of the IMS-based tests in 25 (65.8%) of 38 faeces samples from putative ‘non-infected’ badgers (Fig. 4). This was unexpected since faecal shedding of *M. bovis* by badgers has been generally associated with advanced disease [9], and may be intermittent and heterogeneous [12]. It is possible that some of these putative ‘non-infected’ badgers were misclassified as such owing to limitations in live test performance [31]. Also, in the cases of the IMS-LFD and IMS-qPCR tests, positive results could have arisen from the detection of dead *M. bovis* cells originating from the environment and not indicative of infection, although an IMS-culture test positive result requires mycobacterial growth and so indicates the presence of live viable bacteria. In conclusion, the present study was not designed to provide estimates of the proportion of badgers shedding viable *M. bovis* in their faeces, rather to test the performance of novel non-invasive tests, nevertheless our results suggest that further investigation of the extent of faecal shedding in badgers is warranted.

In this study, *M. bovis* cells were concentrated and selectively captured by IMS from several millilitres of an homogenate of approximately 1 g faeces in PBS in advance of detection by LFD, qPCR and culture during this study; a testing approach that has never been used before on badger faeces. The traditional badger faeces testing approach would be to decontaminate faecal suspensions in PBS with 0.75% cetylpyridinium chloride overnight prior to culture in liquid and/or solid media [10] or to extract total community DNA from approximately 0.1 g faeces prior to *M. bovis*-specific qPCR [12]. By testing a larger quantity of faeces, selectively isolating *M. bovis* cells prior to DNA extraction as well as separating them from PCR inhibitors by IMS, and not employing chemical decontamination which is known to have an adverse impact on the viability of *M. bovis* cells [32, 33], the chances of detection/isolation of *M. bovis* from faeces were likely to have been improved. The three IMS-based methods do not have equivalent detection sensitivity, so it is not surprising that the least sensitive test (IMS-LFD assay) detected lower numbers of *M. bovis* positive faecal samples than both IMS-qPCR and IMS-culture (Fig. 3a and b). Stewart et al. [22] previously reported that IMS-qPCR and IMS-culture improved detection rates of *M. bovis* in cattle lymph nodes compared to direct qPCR and chemical decontamination and culture. Results of the present study suggest that a similar boost in *M. bovis* detection rates has generally been observed by applying these IMS-based methods to test badger faeces. IMS applied to faeces samples in advance of application to the LFD should in theory have improved the detection sensitivity of the LFD, since *M. bovis* cells were concentrated from 6 ml faecal homogenate into 200 ul before testing. In reality, despite the fact that smaller (300 nm) paramagnetic beads were employed, samples after field IMS on faeces samples did not always run easily along the LFD due to bead aggregation. Consequently, detection sensitivity actually decreased and this was not simply due to a sample matrix effect. Results of a subsequent ring trial study to compare IMS-LFD and direct LFD with other methods for detecting *M. bovis* in badger faeces showed that fewer positive results were obtained after IMS than by direct LFD testing [34].

## Conclusions

A novel prototype *M. bovis*-specific LFD was successfully developed which, in combination with IMS, was shown to be capable of detecting *M. bovis* cells in some badger faeces samples tested during this study. However, there was generally little agreement between results of field IMS-LFD and laboratory-based IMS-qPCR and IMS-culture tests; the IMS-LFD assay detected the lowest percentage of *M. bovis*-infected badger faecal samples, reflective of its higher LOD_50%_. The novel IMS-LFD test was shown to have relatively high diagnostic Sp (0.926, 95% CI: 0.837–0.976) but low diagnostic sensitivity (0.081, 95% CI: 0.027–0.178), relative to a suite of other live animal diagnostic tests. In light of these findings, the potential of the novel IMS-LFD test for *M. bovis* in badger faeces as a non-invasive field test for badger surveillance purposes would appear to be potentially limited to detecting badgers shedding high numbers of *M. bovis* (≥10^5^ CFU/g) in their faeces. Alternative applications for the novel *M. bovis*-specific LFD are currently being explored.

## Abbreviations

95% CI: 95% Confidence Interval
CFU: Colony-forming units, expressed per ml or g
C-line: Control line on LFD
DNA: Deoxyribonucleic acid
IFN-γ EIA: Interferon Gamma Enzyme Immuno Assay
IgG: Immunoglobulin G
IMS: Immunomagnetic Separation
IMS-LFD: Immunomagnetic separation followed by lateral flow device test
IMS-culture: Immunomagnetic separation followed by culture in BD MGIT culture medium
IMS-qPCR: Immunomagnetic separation followed by quantitative PCR
LFD: Lateral flow device
LOD_50%_: 50% limit of detection
Mab: Monoclonal antibody
Pab: Polyclonal antibody
PBS: Phosphate buffered saline, pH 7.2
PBS-T: PBS containing 0.05% Tween 20
PCR: Polymerase chain reaction
qPCR: Quantitative PCR
RTA: Road traffic accidents
Se: Diagnostic sensitivity
Sp: Diagnostic specificity
TB: Tuberculosis, in this case bovine tuberculosis
T-line: Test line on LFD
